# Enhanced visualization of mobile chest X-ray images in the intensive care setting using software scatter correction

**DOI:** 10.1177/02841851221087631

**Published:** 2022-03-15

**Authors:** Harry Targett, Dominic Hutchinson, Richard Hartley, Richard McWilliam, Ben Lopez, Ben Crone, Stephen Bonner

**Affiliations:** 1 Department of Clinical Radiology, 5460 South Tees Hospitals NHS Foundation Trust, The James Cook University Hospital, Rd, Middlesbrough, UK; 2 Department of Critical Care, 5460 South Tees Hospitals NHS Foundation Trust, The James Cook University Hospital, Middlesbrough, UK; 3 IBEX Innovations Ltd., Explorer 2, Sedgefield, 3FF, UK

**Keywords:** Digital radiography, mobile chest X-ray, scatter correction, technology assessments

## Abstract

**Background:**

Mobile chest X-ray (CXR) scans are performed within intensive treatment units (ITU) without anti-scatter grids for confirming tube and line hardware placement. Assessment is therefore challenging due to degraded subject contrast resulting from scatter.

**Purpose:**

To evaluate the efficacy of a software scatter correction method (commercially named Trueview) for enhanced hardware visualization and diagnostic quality in the ITU setting.

**Material and Methods:**

A total of 30 CXR scans were processed using Trueview and compared with standard original equipment manufacturer (OEM) images via observer scoring study involving two radiology and four ITU doctors to compare visualization of tubes and lines. Results were analyzed to determine observer preference and likelihood of diagnostic quality.

**Results:**

Reviewers were more likely to score Trueview higher than OEM for mediastinal structures, bones, retrocardiac region, tube visibility, and tube safety (*P* *<* 0*.*01). Visual grading characteristic analysis suggested a clinical preference for Trueview compared with OEM for mediastinal structures (area under the visual grading characteristic curve [AUC_VGC_] = 0.60, 95% confidence interval [CI] = 0.55–0.65), bones (AUC_VGC_ = 0.61, 95% CI = 0.55–0.66), retrocardiac region (AUC_VGC_ = 0.64, 95% CI = 0.59–0.69), tube visibility (AUC_VGC_ = 0.65, 95% CI = 0.60–0.70), and tube safety (AUC_VGC_ = 0.68, 95% CI = 0.64–0.73). Reviewers were indifferent to visualization of the lung fields (AUC_VGC_ = 0.49, 95% CI = 0.44–0.55). Registrars (3/6 reviewers) were indifferent to the mediastinal structure regions (AUC_VGC_ = 0.54, 95% CI = 0.47–0.62).

**Conclusion:**

Reviewers were more confident in identifying the placement and safety of tubes and lines when reviewing Trueview images than they were when reviewing OEM.

## Introduction

Bedside chest X-ray (CXR) radiography is necessary within intensive treatment unit (ITU) wards when the patient cannot be moved to a fixed system (1) and when rapidly evolving conditions must be assessed. A mobile X-ray system is used whereby the source and detector are manually positioned. Recorded images are used for detection of clinical abnormalities (2) and verification of tube and line placements (3,4). Clinical interpretation of mobile CXRs is challenging due to sub-optimal patient positioning, the diverse array of pathologies present in patients admitted to intensive care units (5), and viewing on non-diagnostic display devices. Since anti-scatter grids (ASGs) cannot be used in mobile systems due to practical alignment difficulties, degraded image quality will occur due to relatively increased scatter. There are documented instances of incorrect line and tube placements leading to serious side effects (6,7), most notably for cases where incorrect identification of nasogastric tube position has led to life-threatening complications due to deposition of medications and nasogastric feed into lung rather than the gastrointestinal tract. As a result, enhancing the diagnostic quality of bedside CXRs could provide significant value in the ITU setting.

ASGs are not commonly used for mobile X-ray examinations due to difficulties in achieving consistent and accurate placement. Unless the grid is aligned carefully with respect to the source, a dramatic loss of contrast improvement factor (CIF) occurs (8). This limitation leads to excessive scatter and a degraded discernibility of weak contrast features such as tubes and lines. Novel methods for assisting the process of grid alignment in mobile arrangements have been proposed (9) but these approaches are generally compromised by the need for line of sight not available in bedside setups. As an alternative to ASGs, software scatter correction estimates the spatial variation of background scatter incurred by the patient body composition, which is numerically subtracted from the image. Skyflow (Philips Healthcare, Hamburg, Germany) predicts scatter based on a pre-simulated Monte Carlo database for phantom CXR (10), skeletal imaging (11), and catheter tip visualization for bedside scans (12). SimGrid™ (Samsung Electronics Co. Ltd, Suwon, Republic of Korea) estimates scatter based on a trained convolutional neural network (CNN) for bedside CXR images (13). Similar to Skyflow, SimGrid™ relies upon a training database containing representative patient size and composition. The major limitations of the current methods are as follows: (i) they do not fully account for the actual scattering characteristics of the unique patient or the specific X-ray system; and (ii) machine learning or physics models require extensive training databases. Physics based simulators tend to utilize approximate point spread functions (PSFs) and convolution (14) or else some form of polynomial shape (15). While superior to polynomial methods, PSF methods are limited in the following ways: (i) they are assumed to be isotropic (14), stationary or piecewise stationary (16), or else invariant to the material composition (17); (ii) differences between the PSF approximation and simulator are not considered; and (iii) differences between the simulator and real world are not considered. Hence, the resulting predicted scatter contains large errors across the image field that vary depending on the specific X-ray system and patient size/composition (18). For this study, a novel scatter removal method called Trueview (Ibex Innovations Ltd., Sedgefield, United Kingdom) is employed that uses a physics-based approach to estimate the amount of scatter present according to a model of the DR system built offline (19). The model estimates the X-ray image output for a given body part using non-stationary, non-isotropic PSFs. It should be noted that, unlike ASGs, methods of software scatter removal do not directly address noise due to scatter and therefore noise mitigation must be implemented within the image processing pipeline.

The aim of the present study was to compare the diagnostic image quality of clinical CXR images generated by the Trueview and original equipment manufacturer (OEM) mobile system based on visualization of tube/line hardware and confirmation of safe placement under standard ITU ward viewing conditions. A secondary aim was to comparatively assess the visualization of anatomical features (lungs, retrocardiac region, mediastinum, and bones).

## Material and Methods

A series of 148 ITU ward CXR images were collected from an Agfa (Agfa HealthCare N.V., Mortsel, Belgium) Dx-D100 mobile system in both unprocessed raw and postprocessed DICOM formats during routine activity at The James Cook University Hospital, UK. Patient consent was not sought since the collected data were anonymized raw data with no clinical implications for patients and from which patients could not be identified. X-ray source parameters were within standard mobile CXR protocol (74–90 kVp; 1.92–10.2 mAs).

### Data processing

**Fig. 1.**
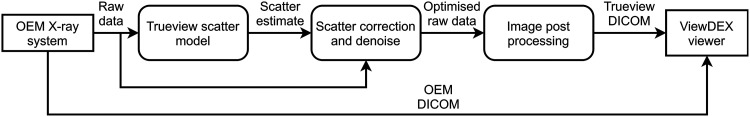
Pipeline flow diagram for Trueview scatter removal and image postprocessing.

**Fig. 2.**
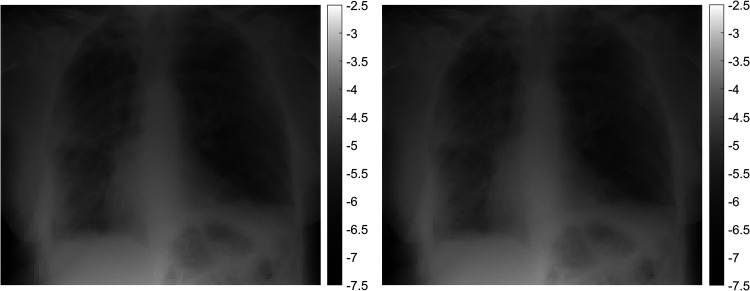
Left: Real X-ray image. Right: Simulated X-ray image from the physics model after making morphological adjustments to hard- and soft-tissue composition via an inverse problem-solving approach. Both images have been down-sampled to a lower spatial resolution by a factor of 23 and intensity displayed in negative log space.

**Fig. 3.**
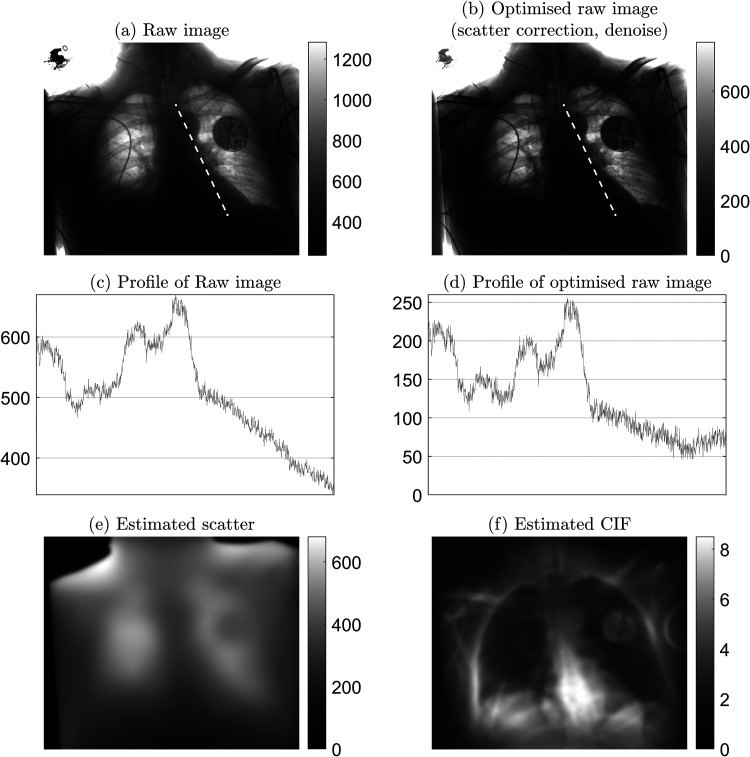
Example of raw image optimization: 2.6 mAs, kVp = 85, exposure index = 297. (a) Raw image as measured at detector. (b) Optimized raw image after applying Trueview scatter correction and strategic denoise. (c) Profile of raw image, showing intensity variation along the retrocardiac region as indicated by the dashed line in (a). (d) Corresponding profile for optimized raw image. (e) Predicted background scatter. (f) Contrast improvement factor (CIF) as a function of applied scatter correction, where higher values indicate regions of greater amplification of local contrast.

Raw images were scatter-corrected and postprocessed using the Trueview image processing pipeline (Fig. 1), resulting in a set of Trueview DICOM images for comparison with the equivalent OEM DICOM images. The starting point for the Trueview inverse problem-solving approach involves certain assumptions about sample composition, such as the overall thickness and position of tissue and bone. The output of a simulated image is compared to the real X-ray image, and the error between simulated and measured images is used to refine the model by applying morphological changes to the digital body part input to the simulator. This comparison is repeated many times for varying thicknesses and proportions of tissue and bone, until the simulated X-ray image and the real X-ray image agree sufficiently that any disagreement cannot be differentiated from the underlying statistical errors in the system. An example visual comparison between the simulated and real X-ray image is shown in Fig. 2, demonstrating the accuracy of the non-stationary non-isotropic approach. The typical compute time for generation of a CXR scatter estimate is 11.4 s (based on an Intel i7-7800X CPU, 32 GB ram with GTX1070 GPU). Subtraction of the predicted estimated scatter leads to physically accurate contrast improvement across the image comparable to or exceeding that of a typical ASG (20). Scatter correction is followed by multiscale iterative denoise (21,22) strategically applied according to the local predicted scatter conditions. An example of predicted scatter, corrected raw image, and contrast improvement for a specific patient is shown in Fig. 3.

**Fig. 4.**
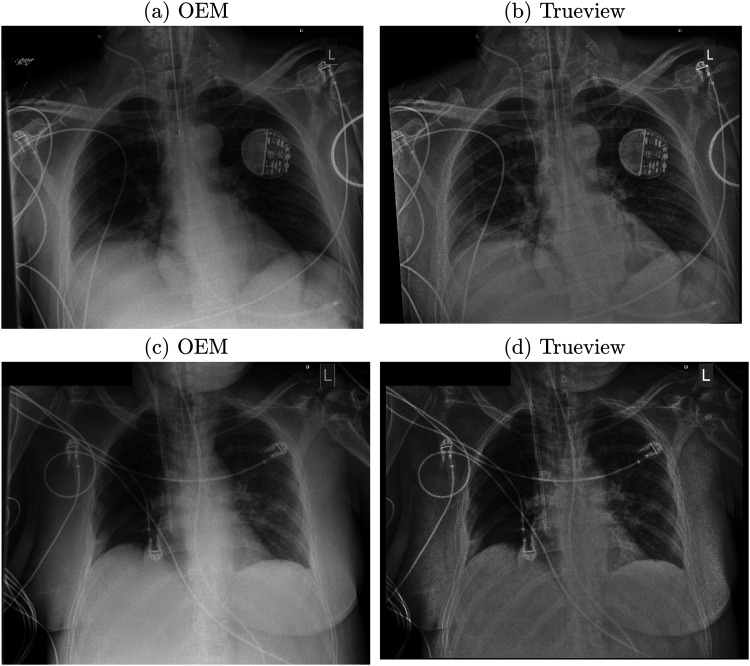
Examples of postprocessed enhanced OEM and Trueview images. (a, b) 2.6 mAs, kVp = 85, exposure index = 297. (c, d) Exposure: 6.4 mAs, kVp = 85, exposure index = 251.

**Fig. 5.**
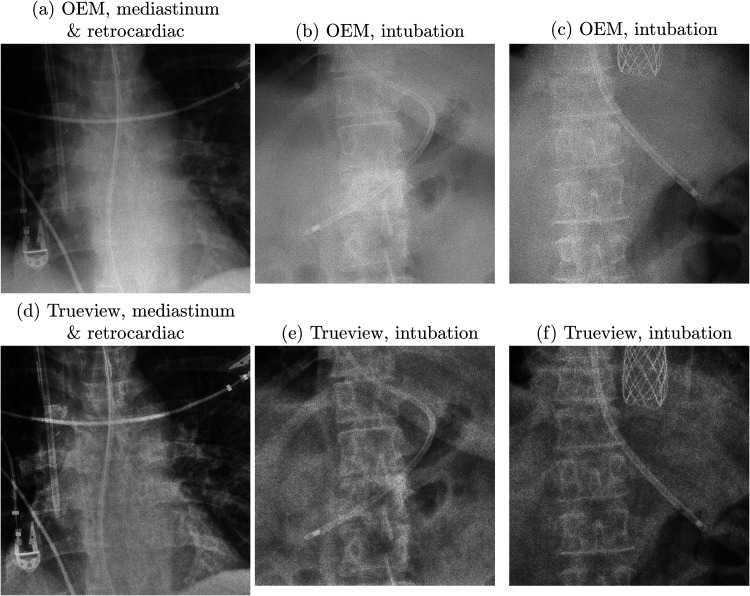
OEM (upper row) and Trueview (lower row) images compared on the basis of visualization of anatomical features and nasogastric tube. (a, d) 6.4 mAs, kVp = 85, exposure index = 251. (b, e) 3.8 mAs, kVp = 88, exposure index = 434. (c, f) 2.6 mAs, kVp = 85, exposure index = 250.

Corrected raw images are postprocessed for diagnostic viewing using a multiscale contrast enhancement pipeline (23) that includes strategic contrast and noise management for the lung field and mediastinum. OEM overlay graphics were replicated within the Trueview image to minimize visual differences. Examples of Trueview outputs are illustrated in Fig. 4, where increased subject contrast can be seen in comparison to the corresponding OEM images. Further comparisons of cropped regions are shown in Fig. 5. The assembled observer scoring image set was created by shortlisting 30 Trueview and 30 corresponding OEM DICOM images according to the elimination criteria described in Table 1.

**Table 1.** Criteria for elimination of images from acquired set.

Stage	Elimination criteria	Ne*
1	No nasogastric or endotracheal tube present	59
2	Non-standard AP CXR view	49
3	Poor image quality occurring in OEM and/or Trueview	10

*The number of images eliminated by each respective elimination stage.

AP, anteroposterior; CXR, chest X-ray; OEM, original equipment manufacturer.

### Image assessment

Observer scoring was based on visual grading criteria comprising four questions themed on diagnostic quality and two themed on visualization and confirmation of tube and line positioning (Table 2). A 5-point rating scale was used to rate observer confidence in fulfilling the scoring criteria (Table 3). Images were assessed in blind, randomized order by each observer using the ViewDEX tool (24). A standard laptop (15.6 inch, 1920 × 1080 resolution, set to 100% brightness) was used to resemble real-world ITU ward viewing conditions. The image set was supplemented by eight random duplicate images (comprising four OEM and four Trueview) for the purpose of analyzing reviewer consistency, resulting in 68 images reviewed by each observer. Six observers were recruited from ITU (two consultants, two registrars) and radiology (one consultant, one registrar). Reviews were conducted in the same location with standard lighting and no direct sunlight.

**Table 2.** Observer scoring questions.

	Visual grading characteristic
Q1	Lungs are clearly visualized and sufficient for diagnostic purposes
Q2	Retrocardiac region is clearly visualized and sufficient for diagnostic purposes
Q3	Mediastinum is clearly visualized and sufficient for diagnostic purposes
Q4	Bones are clearly visualized and sufficient for diagnostic purposes
Q5	All tube/line/wires can be clearly identified
Q6	Positioning of tubes/lines/wires can be confirmed to be safe with high confidence

**Table 3.** Five-point rating scale used for observer scoring. N/A = not applicable.

Scale	Assessment	VGC scale
(a)	Strongly disagree	1
(b)	Somewhat disagree	2
(c)	Neither agree nor disagree	3
(d)	Somewhat agree	4
(e)	Strongly agree	5
N/A	Not applicable	N/A

N/A, not applicable; VGC, visual grading characteristic.

### Statistical analysis

The rating scale shown in Table 3 was interpreted to correspond to a visual grading characteristics (VGC) scale of 1–5 (25). Trueview and OEM images were paired and the mean difference (Trueview minus OEM) between paired scores reported. For cases where two reviews exist for one image, the average score was used to calculate the difference, yielding a sample size of 30 OEM-Trueview pairs evaluated by six observers according to the visual grading criteria. A total of two N/A responses were reported (representing 0.08% of all scores) and were removed from the analysis. To analyze whether the population mean of the differences are different from zero, a paired t-test *P* value was calculated, under the null hypothesis that the mean of the difference is zero (26). To analyze whether the distribution of scores is different, the statistical analysis follows an analogous approach (11). The following two measures were used: (i) a Wilcoxon *P* value was calculated (under the null hypothesis that the distribution of the pairs is symmetric about zero) (27); and (ii) the area under the visual grading characteristic curve (AUC_VGC_) was reported along with a 95% confidence interval (CI), which was calculated using a non-parametric boot strap with 5000 samples (28, 29). If all three measures indicate the same preference for either Trueview or OEM at a 5% significance level, we regarded this as strong evidence that the reviewers’ responses indicated a preference for either Trueview or OEM. The analysis was repeated for all reviewers, consultants, registrars, radiologists, and ITU doctor.

We define a diagnostically acceptable region when the score for a region is ≥4. To assess the likelihood of diagnostic acceptability, a logistic regression model was used, regressing Trueview (along with an intercept) against whether the score for a region is ≥4. The exponential of the parameter estimates for Trueview and the 95% CI are reported. This analysis was performed for all reviewers together. To test the temporal stability of reviewer scoring, Spearman's correlation (*ρ*) and the *P* value under the null hypothesis that *ρ* = 0 are reported. To test inter-reviewer consistency, a two-way random, consistency, single measure analysis was used, and we reported the interclass correlation coefficient (ICC) with associated 95% CI. This analysis was repeated for all scores, Trueview and OEM. Analysis was carried out using the R statistical package (R Foundation, Vienna, Austria) (30).

## Results

**Fig. 6.**
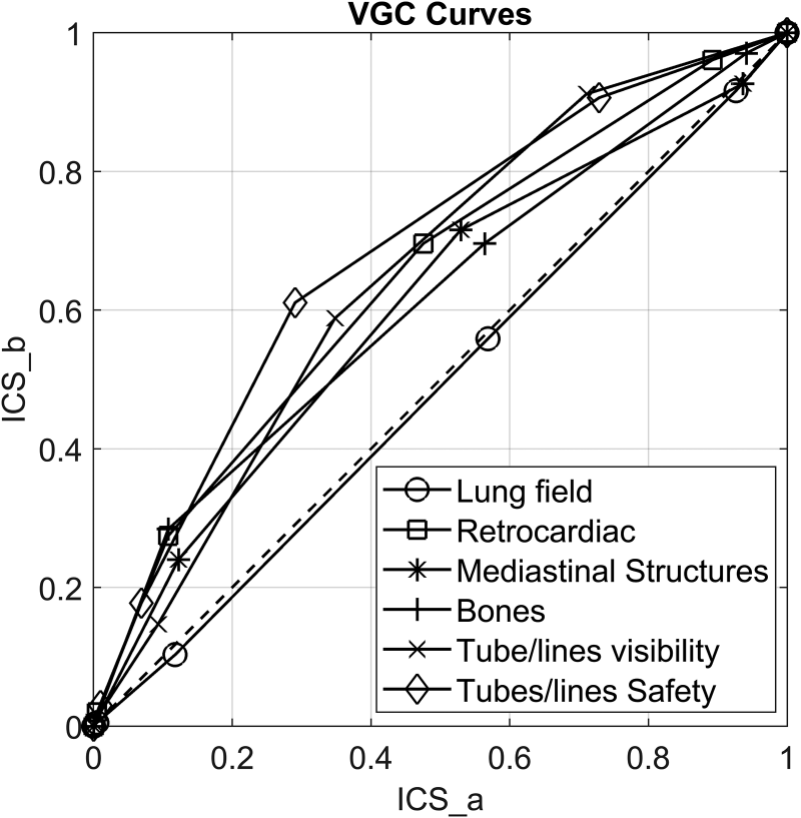
Plot showing area under visual grading characteristic (VCG) curves. ICS_a = image criteria score relating to OEM; ICS_b = image criteria score relating to Trueview.

Statistical analysis of the difference between observer scoring for Trueview and OEM is summarized in Table 4, broken down into each scoring criteria and considering all reviewers. In terms of diagnostic image quality, there is no evidence to suggest that Trueview was rated significantly lower than OEM for any criteria. There is strong evidence for higher preference for Trueview considering criteria Q2–Q6, while Q1 shows no evidence of a preference. Hence, observers were indifferent to the region of the lung fields but preferred diagnostic presentation of Trueview for mediastinal structures, bones, and retrocardiac region. The AUC_VGC_ curves for the lung and retrocardiac regions are illustrated for comparison in Fig. 6. The AUC for lungs is close to 0.5 (0.49) while being significantly higher for the retrocardiac region (0.64). This is further confirmed by the upper/lower 95% confidence limits for retrocardiac region being significantly >0.5. Similar VGC outcomes are apparent for bone and mediastinal structure regions.

**Table 4.** Statistical analysis of difference between observer scores (Trueview − OEM) for each scoring criteria and considering all observers.

	MD ± SD	n	t-*P*(*z*|*H*)	W-*P*(*z*|*H*)	AUC_VGC_	95% CI
*All reviewers*						
Lung fields	−0.02 ± 0.86	204	0.78	0.79	0.49	0.44–0.55
Retrocardiac	0.48 ± 0.87	204	<0*.*01	<0*.*01	0.64	0.59–0.69
Mediastinal structures	0.27 ± 0.80	204	<0*.*01	<0*.*01	0.60	0.55–0.65
Bones	0.36 ± 0.83	204	<0*.*01	<0*.*01	0.61	0.55–0.66
Tube visibility	0.50 ± 0.86	204	<0*.*01	<0*.*01	0.65	0.60–0.70
Tube safety	0.61 ± 0.96	203	<0*.*01	<0*.*01	0.68	0.64–0.73
*Consultants*						
Lung fields	−0.01 (0.98)	102	0.92	0.98	0.50	0.43–0.58
Retrocardiac	0.53 (1.04)	102	<0*.*01	<0*.*01	0.66	0.59–0.73
Mediastinal structures	0.46 (0.81)	102	<0*.*01	<0*.*01	0.67	0.60–0.74
Bones	0.34 (0.95)	102	<0*.*01	<0*.*01	0.61	0.53–0.67
Tube visibility	0.35 (0.92)	102	<0*.*01	<0*.*01	0.61	0.53–0.68
Tube safety	0.58 (1.09)	102	<0*.*01	<0*.*01	0.66	0.58–0.72
*Registrars*						
Lung fields	−0.02 (0.73)	102	0.73	0.70	0.48	0.41–0.55
Retrocardiac	0.42 (0.67)	102	<0*.*01	<0*.*01	0.62	0.50–0.69
Mediastinal structures	0.09 (0.75)	102	0.21	0.23	0.54	0.47–0.62
Bones	0.38 (0.71)	102	<0*.*01	<0*.*01	0.61	0.53–0.68
Tube visibility	0.64 (0.78)	102	<0*.*01	<0*.*01	0.69	0.62–0.76
Tube safety	0.63 (0.82)	101	<0*.*01	<0*.*01	0.71	0.64–0.78
*Radiologists*						
Lung fields	0.09 (0.66)	68	0.28	0.28	0.55	0.45–0.63
Retrocardiac	0.56 (0.75)	68	<0*.*01	<0*.*01	0.69	0.59–0.76
Mediastinal structures	0.54 (0.75)	68	<0*.*01	<0*.*01	0.72	0.63–0.78
Bones	0.64 (0.79)	68	<0*.*01	<0*.*01	0.72	0.64–0.79
Tube visibility	0.49 (0.61)	68	<0*.*01	<0*.*01	0.69	0.60–0.76
Tube safety	0.69 (0.71)	68	<0*.*01	<0*.*01	0.77	0.69–0.83
*ITU doctor*						
Lung fields	−0.07 (0.94)	136	0.39	0.39	0.47	0.40–0.53
Retrocardiac	0.43 (0.93)	136	<0*.*01	<0*.*01	0.63	0.57–0.69
Mediastinal structures	0.14 (0.80)	136	0.04	0.05	0.56	0.50–0.63
Bones	0.22 (0.82)	136	<0*.*01	<0*.*01	0.56	0.49–0.62
Tube visibility	0.50 (0.97)	136	<0*.*01	<0*.*01	0.64	0.57–0.70
Tube safety	0.56 (1.07)	135	<0*.*01	<0*.*01	0.65	0.59–0.71

AUC_VGC_, area under the visual grading characteristic curve; CI, confidence interval; MD, mean difference; OEM, original equipment manufacturer; SD, standard deviation; t-*P*(*z*|*H*), *t*-test *P* value; W-*P*(*z*|*H*), Wilcoxon signed-rank test *P* value.

The results for temporal stability are given in Table 5. There is some evidence for non-zero Spearman's correlation for the lungs and bones, the latter being more statistically compelling. Although some drift may be present for some questions, it is low level and the difference between Trueview and OEM is not statistically significant. Therefore, the drift is unlikely to confound the comparison of the two methods.

**Table 5.** Results from statistical analysis of temporal drift in scores.

	All	Trueview	OEM
Lung fields	0.1 (0.04)	0.08 (0.24)	0.11 (0.12)
Retrocardiac	0.06 (0.2)	0.15 (0.03)	0.03 (0.67)
Mediastinal structures	−0.06 (0.19)	−0.08 (0.23)	−0.02 (0.81)
Bones	−0.17 (<0.01)	−0.08 (0.23)	−0.22 (<0.01)
Tube visibility	0.01 (0.79)	0.04 (0.57)	0.03 (0.67)
Tube safety	−0.01 (0.85)	−0.01 (0.86)	0.05 (0.51)

Values are given as Spearman's correlation *ρ* (with associated *P* value under the null hypothesis that *ρ* = 0).

OEM, original equipment manufacturer.

The results for the statistical analysis of consistency are shown in Table 6. The consistency for tube visibility and tube safety was higher than for the image quality questions. The difference in consistency between the questions is close to being within statistical error but there is some evidence that responses for the lung fields region were less consistent than tube visibility and tube safety. As there is little evidence of a difference in consistency between Trueview and OEM, the comparison is not confounded by reviewer consistency.

**Table 6.** Results from statistical analysis of inter-reviewer consistency.

	All	Trueview	OEM
Lung fields	0.6 (0.44–0.73)	0.69 (0.5–0.83)	0.49 (0.16–0.71)
Retrocardiac	0.78 (0.69–0.85)	0.68 (0.47–0.82)	0.78 (0.64–0.88)
Mediastinal structures	0.63 (0.47–0.75)	0.61 (0.37–0.78)	0.59 (0.34–0.77)
Bones	0.66 (0.52–0.77)	0.55 (0.27–0.75)	0.69 (0.5–0.83)
Tube visibility	0.82 (0.75–0.88)	0.8 (0.68–0.89)	0.8 (0.68–0.89)
Tube safety	0.86 (0.8–0.91)	0.86 (0.77–0.92)	0.83 (0.72–0.91)

Values are given as interclass correlation coefficient (95% confidence interval).

In terms of the likelihood of landmark regions being of diagnostic quality, the logistic regression confirmed no significant preferences for Trueview in the lung fields region: responses were 0.94 times (95% CI = 0.65–1.42) more likely to be agree that the region was diagnostic quality for Trueview compared with OEM. However, preference for Trueview for other landmark regions was again confirmed:

– Responses were 2.92 times (95% CI = 1.69–3.80) more likely to agree the retrocardiac region was of diagnostic quality in Trueview than with OEM.
– Responses were 2.23 times (95% CI = 1.48–3.38) more likely to agree the mediastinal structures region was of diagnostic quality in Trueview than with OEM.
– Responses were 1.77 times (95% CI = 1.18–2.67) more likely to agree the bones region was of diagnostic quality in Trueview than with OEM.

In terms of tube and line visualization, there was also strong evidence for observer preference for Trueview considering Q5 and Q6. Observers therefore had higher confidence in identifying hardware and confirming safe placement when viewing the images using a non-specialist display as typically used in ITU wards. The AUC_VGC_ curves for tube and line visibility and placement confirmation are illustrated in Fig. 6 where again strong observer preference for Trueview can be seen. In terms of likelihood of images being of diagnostic quality, responses were as follows:

– 2.67 times (95% CI = 1.80–4.01) more likely to agree (score ≥4) that lines and tubes could be visualized with Trueview images than with OEM; and
– 3.83 times (95% CI = 2.54–5.83) more likely to agree that lines and tubes could be confirmed to be safe with Trueview images than with OEM.

## Discussion

Observer scoring indicated an overall preference for Trueview for the mediastinum, bone, and retrocardiac landmarks, suggesting that underlying diagnostic quality is improved when viewing enhanced Trueview images. Registrar observers were relatively indifferent to the mediastinal structure region compared to a clear preference among consultants. This may be explained by higher contrast improvement in the mediastinum region (Fig. 3f) and therefore enhanced uniformity of local contrast to background (Figs. 4 and 5) compared to OEM. Such differences may require more opportunity for the registrars to become familiarized with the Trueview presentation. A further possible influence is that ITU doctors are generally less familiar with viewing low scattering, grid-less images in comparison to radiologists. Observers were consistently indifferent to the lung fields region – a region of relatively low contrast improvement and therefore less impacted by scatter correction. As seen in Fig. 4, the ribs and tissue contrast are subtly influenced but less critical when viewing in ITU ward conditions. Hence viewing via medical-grade screen would be necessary to determine radiological value. In the skeletal grid-less study by Lisson et al. (11), a similar scoring methodology within the trauma room setting was adopted but focusing on bone features. Direct comparison with our study is, however, challenging due to the use of a medical-grade viewing screen and visual grading criteria based on visually sharp reproduction. The mobile bedside study by Ahn et al. (13) compared “non-grid” versus “grid-like” scatter-corrected chest images viewed side-by-side using a scoring criterion based on observer preference and hence a direct comparison cannot be drawn.

A strong overall preference for tube and line visualizations and confidence in assessing safe placement is consistent with the assumption that critical hardware is likely to coincide with highly scattering mediastinal structures and abdominal soft tissues, where the subject contrast of intubation features is enhanced through scatter correction and postprocessing. The overall observer impression of tube and line visibility is assumed to be influenced by the chosen combination of viewing conditions, scatter removal, and image postprocessing. While postprocessing settings were standardized across all images, the pipeline itself could be refined to further balance contrast improvement and diagnostic presentation. Noise management in the mediastinum appears effective, noting that ASGs additionally absorb noise due to scatter (16).

In conclusion, there was evidence that Trueview scatter correction increases clinicians’ confidence in reviewing chest images within an ITU setting. Visualization of scatter-sensitive regions (mediastinal structures, retrocardiac region) was preferred for Trueview along with tube visibility and tube safety. The results suggest that Trueview software scatter removal combined with optimized image postprocessing brings viewing contrast benefits, exploiting the scatter estimation sensitivity to patient size and composition as well as X-ray system parameters. Observers were relatively indifferent to the presented lung fields region, confirming that Trueview image processing retains a familiar presentation while enhancing visualization of the scatter-sensitive regions and intubation hardware. Enhancing the ability of clinicians to identify correctly placed tubes enhances patient safety reduces the likelihood of never events and should reduce unnecessary repeat radiographs from being performed. Further research is needed to evaluate the radiological value beyond the ITU setting and for a wider range of patient size and composition.
